# A Quality Improvement Emergency Department Surge Management Platform (SurgeCon): Protocol for a Stepped Wedge Cluster Randomized Trial

**DOI:** 10.2196/30454

**Published:** 2022-03-24

**Authors:** Hensley H Mariathas, Oliver Hurley, Nahid Rahimipour Anaraki, Christina Young, Christopher Patey, Paul Norman, Kris Aubrey-Bassler, Peizhong Peter Wang, Veeresh Gadag, Hai V Nguyen, Holly Etchegary, Farah McCrate, John C Knight, Shabnam Asghari

**Affiliations:** 1 Centre for Rural Health Studies, Faculty of Medicine Memorial University of Newfoundland St. John's, NL Canada; 2 Faculty of Medicine Memorial University of Newfoundland St. John's, NL Canada; 3 Eastern Health Carbonear Institute for Rural Reach and Innovation by the Sea, Carbonear General Hospital Carbonear, NL Canada; 4 Dalla Lana School of Public Health University of Toronto Toronto, ON Canada; 5 Division of Community Health and Humanities, Faculty of Medicine Memorial University of Newfoundland St. John's, NL Canada; 6 School of Pharmacy Memorial University of Newfoundland St. John's, NL Canada; 7 Department of Research and Innovation Eastern Health St. John's, NL Canada; 8 Newfoundland and Labrador Centre for Health Information St. John's, NL Canada

**Keywords:** SurgeCon, emergency department, stepped wedge design, cluster randomized trials, wait time

## Abstract

**Background:**

Despite many efforts, long wait times and overcrowding in emergency departments (EDs) have remained a significant health service issue in Canada. For several years, Canada has had one of the longest wait times among the Organisation for Economic Co-operation and Development countries. From a patient’s perspective, this challenge has been described as “patients wait in pain or discomfort for hours before being seen at EDs.” To overcome the challenge of increased wait times, we developed an innovative ED management platform called *SurgeCon* that was designed based on continuous quality improvement principles to maintain patient flow and mitigate the impact of patient surge on ED efficiency. The SurgeCon quality improvement intervention includes a protocol-driven software platform, restructures ED organization and workflow, and aims to establish a more patient-centric environment. We piloted SurgeCon at an ED in Carbonear, Newfoundland and Labrador, and found that there was a 32% reduction in ED wait times.

**Objective:**

The primary objective of this trial is to determine the effects of SurgeCon on ED performance by assessing its impact on length of stay, the time to a physician’s initial assessment, and the number of patients leaving the ED without being seen by a physician. The secondary objectives of this study are to evaluate SurgeCon’s effects on patient satisfaction and patient-reported experiences with ED wait times and its ability to create better-value care by reducing the per-patient cost of delivering ED services.

**Methods:**

The implementation of the intervention will be assessed using a comparative effectiveness-implementation hybrid design. This type of hybrid design is known to shorten the amount of time associated with transitioning interventions from being the focus of research to being used for practice and health care services. All EDs with 24/7 on-site physician support (category *A* hospitals) will be enrolled in a 31-month, pragmatic, stepped wedge cluster randomized trial. All clusters (hospitals) will start with a baseline period of usual care and will be randomized to determine the order and timing of transitioning to intervention care until all hospitals are using the intervention to manage and operationalize their EDs.

**Results:**

Data collection for this study is continuing. As of February 2022, a total of 570 randomly selected patients have participated in telephone interviews concerning patient-reported experiences and patient satisfaction with ED wait times. The first of the 4 EDs was randomly selected, and it is currently using SurgeCon’s eHealth platform and applying efficiency principles that have been learned through training since September 2021. The second randomly selected site will begin intervention implementation in winter 2022.

**Conclusions:**

By assessing the impact of SurgeCon on ED services, we hope to be able to improve wait times and create better-value ED care in this health care context.

**Trial Registration:**

ClinicalTrials.gov NCT04789902; https://clinicaltrials.gov/ct2/show/NCT04789902

**International Registered Report Identifier (IRRID):**

DERR1-10.2196/30454

## Introduction

### Background

Long wait times and overcrowding are challenging emergency departments (EDs) around the world [1-4]. Several other countries with advanced health care systems cannot keep pace with patient demand. In particular, Canada ranks among countries with the longest wait times compared with those of peer-industrialized countries [5]. The Canadian Institute for Health Information (CIHI) reported an 11% increase in ED wait times from 2015-2016 to 2016-2017 [2]. This translates to long wait times and deters patients from pursuing the necessary care they need and increases the likelihood of patients leaving the ED without being seen (LWBS) by a physician [6,7]. In Newfoundland and Labrador, Canada’s easternmost province, long wait times are plaguing the province much like the rest of Canada [8-10].

The Newfoundland and Labrador provincial government and the province’s 4 regional health authorities (RHAs) have the option of expanding the health care workforce [11,12] at a time of historic fiscal restraint or finding effective interventions to improve the efficiency of their current ED service [13]. Overtime [14-16], expanding the ED [17,18] and redirecting patients to primary care [19,20] have not been shown to be effective. According to an October 2020 report from the CIHI, ED services are making up a larger percentage of total hospital spending with a 4% annual growth rate, which was observed between 2005 and 2019 [21]. The same report states that ED staff are twice as likely to work overtime compared with staff in other departments [21].

We have created a quality improvement intervention called SurgeCon. As a pragmatic ED management platform, SurgeCon includes 3 separate components (described below) that together act to decrease wait times and improve the sustainability of Newfoundland and Labrador’s ED services without significant workforce changes. These interventions include restructuring the ED organization and workflow, fostering a patient-centric environment, and quantifying ED demands and available resources in real time. SurgeCon is designed to enable frontline health care workers to anticipate and mitigate surges in patient volume through a series of proactive steps and decision-making tools. SurgeCon attracted the attention of the Newfoundland and Labrador Eastern Health (EH) RHA after they missed their own ED wait time benchmarks in 2016 [22]. The initial development of the SurgeCon intervention came about after an external review was completed by an independent third party to determine which areas of ED operations could be adjusted to improve wait times and departmental efficiency. The external review was one of many components included in a provincial wait time reduction initiative [23].

We piloted SurgeCon at the ED located in Carbonear, Newfoundland and Labrador, an EH administered hospital, over 45 months from July 1, 2013, to March 31, 2017. Data from the pilot study were analyzed using an interrupted time series analysis to assess its effect on ED performance. The resulting change in indicators was noteworthy, despite a 25.7% increase in patient volume. Over the course of the 45-month pilot study, average time to physician’s initial assessment (PIA) decreased from 104.3 (SD 9.9) minutes to 42.2 (SD 8.1) minutes, length of stay (LOS) in the ED decreased from 199.4 (SD 16.8) minutes to 134.4 (SD 14.5) minutes, and the number of patients LWBS decreased from 12.1% (SD 2.2%) to 4.6% (SD 1.7%). All of these changes were statistically significant. The marked and sustained impact of SurgeCon on ED performance in Carbonear supports the case for its extension to other EDs.

The proposed innovative clinical trial and the implementation of SurgeCon (see the implementation paper) [24] will generate practical information on its effectiveness in a range of urban and rural ED settings and generate data to support its more comprehensive implementation. Given the successful results from the pilot study, and if SurgeCon proves to address ED patient flow issues in this study, the rest of Canada and other countries could significantly benefit from its implementation.

Given the scope of the SurgeCon research program, a separate protocol related to evaluating the intervention’s implementation was published in a separate article [24]. This research protocol focuses on the innovative clinical trial stepped wedge cluster randomized trial (SW-CRT) design used to assess the effectiveness of the intervention. In this protocol paper, we will provide a brief description of the intervention and associated research activities that will be carried out at each of the 4 selected ED sites.

### Study Aim and Objectives

In this study, we present the protocol for a 31-month SW-CRT trial. Our aim is to evaluate the performance of the SurgeCon platform in improving important patient and service process outcomes in EDs in an RHA in Newfoundland and Labrador and develop strategies to promote its scalability, sustainability, and successful implementation across the Canadian health system. The primary objective of the trial is to evaluate the effects of SurgeCon on ED performance by assessing its impact on LOS, PIA, and LWBS. The secondary objective of the trial is to assess the intervention’s impact on patient satisfaction and patient-reported experience with ED wait times and its ability to create better-value ED services by reducing ED costs.

## Methods

### Ethics Approval

This study has been approved by the Newfoundland Labrador Health Research Ethics Board with researcher portal file 20201482.

### Study Setting

Newfoundland and Labrador’s health care system is delivered through four RHAs: EH, Central Health, Western Health, and Labrador-Grenfell Health. The eastern RHA will be participating in this research initiative as a collaborative research partner and will be the only RHA participating in the study. The other 3 health authorities will act as knowledge users and will not be included in the SurgeCon trial. However, the research team will provide interim reports to the other health authorities to allow them to monitor and learn from research findings over the course of the study period to guide future implementation in their own centers.

**Figure 1:**
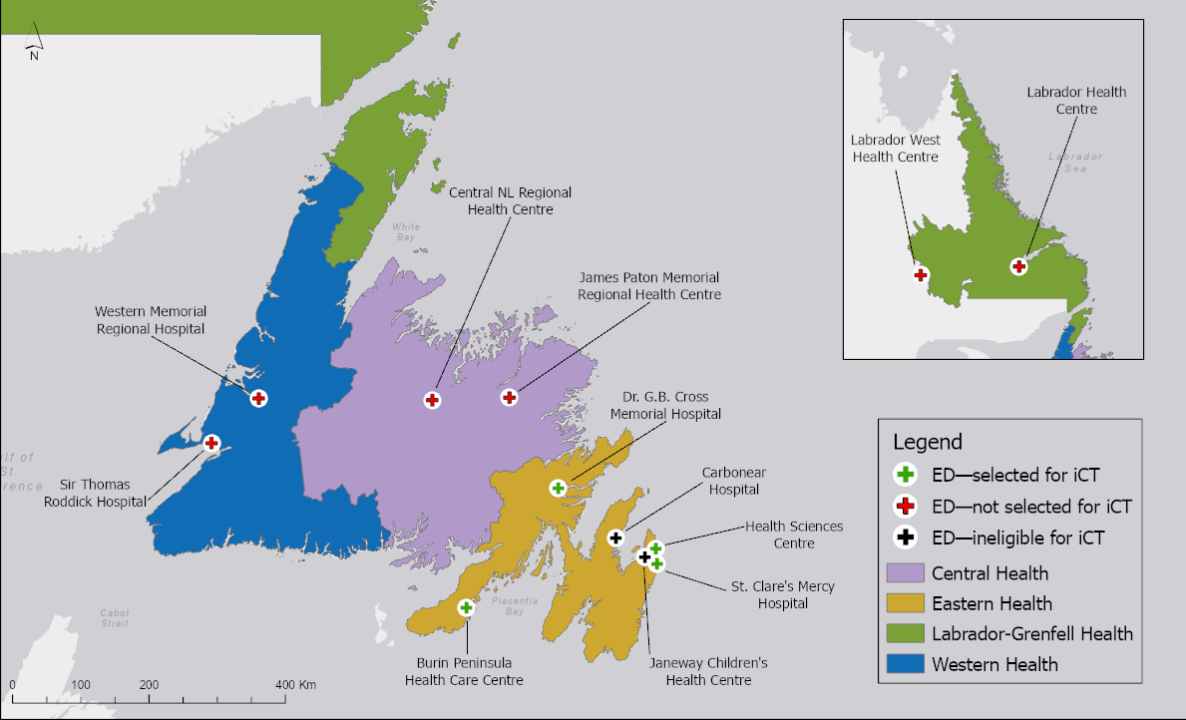
Emergency department (ED) locations in 4 regional health authorities of Newfoundland and Labrador (NL). iCT: innovative clinical trial.

This is a multisite study, including 2 urban and 2 rural EDs with 24/7 on-site physician support in the EH region of Newfoundland (Figure 1). The remaining 2 EDs with 24/7 on-site physician support within the study area that are not receiving the intervention are a pediatric ED, which operates differently and has wait times that differ greatly from adult or general ED, and the Carbonear General Hospital, which was chosen for the pilot study. The 4 sites receiving the intervention include 2 urban sites (Health Sciences Centre and St. Clare’s Mercy Hospital) and 2 rural sites (Dr. G.B. Cross Memorial Hospital and Burin Peninsula Health Care Centre).

### Study Population

All individuals who visited any of the 4 selected EDs during the study period will be included in the collection of deidentified health administrative data. We will also collect and monitor ED-level key performance indicator (KPI) data such as LOS, PIA, and LWBS because they are impacted by patient volume. Patients who receive care at these EDs will be randomly selected for subsequent follow-up after they are discharged to collect information related to their experience in the ED and to determine their level of satisfaction with the care that they received.

### Site Randomization

As we will be evaluating the effectiveness of SurgeCon using an SW-CRT, the 4 different hospitals will be randomized, with 1 ED site starting at the first sequence. The next site will start 6 months later in the second sequence and will continue until all EDs are allocated to sequences. A simple random sample will be performed by the study statistician, who will generate a randomization list using statistical software that will determine the order of intervention implementation. The researchers and participants will not be blinded to whether they are in the intervention or the control cohort.

### Patient Randomization

To assess patient-reported experiences and satisfaction, discharged patients who are subsequently contacted to complete satisfaction and patient experience surveys will be randomly selected using a random time and date generator program.

### Study Outcomes

The SurgeCon study aims to measure both ED KPIs (LOS, PIA, and LWBS) and patient perceptions and satisfaction related to the care they received in the ED. Patient health outcomes, use of health care resources, and overall cost will also be assessed based on a patient’s arrival time. We will also collect information on the potential adverse effects of the intervention such as 24-hour readmission and mortality. These data will help examine trends in mortality and readmissions before and after intervention implementation.

The study uses a comparative effectiveness-implementation hybrid design and includes outcomes for effectiveness and implementation. Two measurement levels will be considered for the study, ED- or service-level and patient-level outcomes will be used to determine the effectiveness of SurgeCon. To guide our choice of outcomes and to determine how best to evaluate the intervention’s implementation, we applied a combination of 2 frameworks, that is, RE-AIM (reach, effectiveness, adoption, implementation, and maintenance) and Consolidated Framework for Implementation Research [25,26]. To guide our implementation strategy, we will collect data related to organizational climate, fidelity of staff training, fidelity of intervention delivery, implementation cost, barriers and enablers to adoption, implementation, institutionalization, intervention acceptability, intervention appropriateness, feasibility of maintaining SurgeCon, sustainability of SurgeCon, and scalability of the SurgeCon intervention. Detailed information on implementation assessment and outcomes is available via our other work [24].

### Study Design

**Figure 2:**
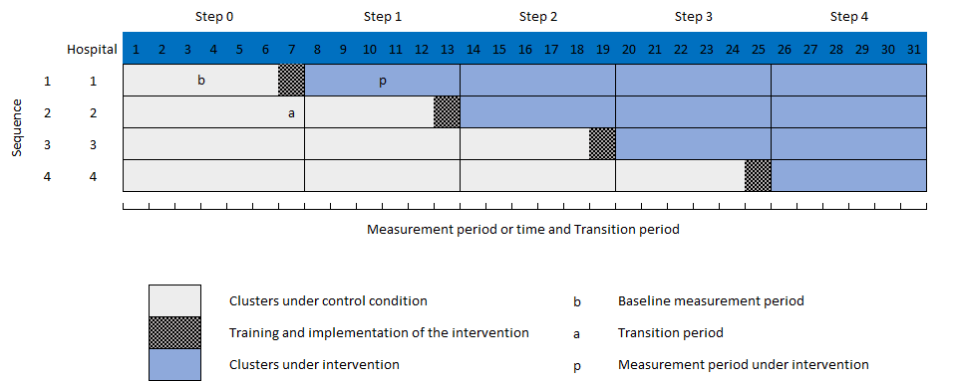
Schematic representation of a SW-CRT with 4 steps for a 31-month study period. SW-CRT: stepped wedge cluster randomized trial.

The SW-CRT design is a novel, robust, and flexible 1-way crossover cluster randomized trial design increasingly being used in trial arms with varying time delays in which all clusters start from the control condition to the active intervention condition state. In particular, this longitudinal stepped wedge study design includes a repeated cross-sectional design, as illustrated in Figure 2, where each hospital will eventually receive the intervention. All participating sites will begin the trial in a control condition where they will continue to use a usual care model or the model of care provided before the beginning of the trial. Each site will switch from providing usual care to providing care using the intervention that will take place at predetermined time periods during the study. At the end of the trial experiment, each of the sites will have implemented all of the intervention’s components. The stepped wedge randomized trial design used in this study is normally carried out at the cluster level rather than at the individual level. Therefore, a clustered randomized stepped wedge design will be the focus of this protocol.

The advantages of the SW-CRT include logistical flexibilities, efficiencies in terms of power, and sample size compared with traditional (clustered) parallel-group designs. Furthermore, the ethical advantages in longitudinal and open cohort studies have also been recognized [27-29]. A simple random sampling technique will be used to determine the order of intervention implementation across the 4 selected sites. The assignment of 1 cluster per sequence will maximize statistical power. The study period can be subdivided by four 6-month steps. Each step starts with the implementation of the intervention at one of the 4 selected sites. Observations are collected repeatedly from each cluster in multiple periods. The key parameters such as the number of clusters or clusters per sequence, steps, and measurement periods (both control and intervention conditions), intracluster correlation coefficient (ICC), and effect size are required for sample size calculation for the SW-CRT design.

The total study period available for the intervention to remain active is 31 months, and it is expected that at least one month will be needed for training and implementation at each ED site. A 1-month intervention adoption period was considered to be sufficient for the intervention to be fully operationalized for a single cluster. During the first 6 months of the baseline period, all patients who visit any of the 4 hospitals will receive usual care. After the baseline period, a randomly allocated hospital will begin SurgeCon intervention implementation every 6 months and will continue to use the intervention for operations and management until the end of the study. Staff at each of the randomly selected hospitals will undergo training, establish processes and guidelines that are consistent with efficiency principles covered during training, and begin routine data entry. During the last 6 months of the study period, all 4 hospitals will be operating using the SurgeCon ED management platform exclusively. We will be monitoring the characteristics of each hospital and the composition of their associated frontline teams to determine if significant changes have occurred since the control period and whether an adjustment to our analysis plan is required.

### Sample Size

On the basis of the results of a pilot study conducted at a rural ED site in EH’s jurisdiction, we established a common framework for optimal sample size calculations when the number of clusters available for randomization is limited to 4 sites for the SW-CRT design. We chose 1 cluster, which will cross over to an intervention state at a randomly assigned step. To keep things relatively simple, we assume that an equal number of observations is sampled in each cross section of each cluster. Alternatively, we expect an equal amount of observations per period per cluster. Results from our pilot study show a 15% to 30% decrease in the LOS or wait times between 6 and 30 months after SurgeCon implementation [30-32]. This trial is powered to detect a 15% change in LOS at 5% of type I error and 80% power with an ICC [33] of 0.1 for repeated measures (6 measures per step). A 10% reduction in ED wait time in the study sites could result in 10-minute reduction in LOS. To detect this change, a minimum sample size of N=20,280 (169/month/hospital for 30 months and 4 hospitals) is required. To be able to conduct age, sex, and patient acuity subgroup analyses for all ED visits across the 4 intervention sites, we will include all ED visits for this portion of the analysis as there is a low cost associated with extracting data from existing ED repositories, which routinely capture patient record-level information. For patient satisfaction, the study is powered to detect a 30% change in patient-reported experience measures or patient satisfaction [34-37] at a 5% of type I error and 80% power with an ICC of 0.1. Therefore, a sample size of 1320 (11/month/hospital for 30 months and 4 hospitals) would be sufficient for this study. Considering a 50% response rate, we will conduct 25 surveys per month per hospital. In particular, we demonstrated the required sample size and power calculation procedure with illustration for different combinations of the ICC to detect the standard effect size. For this illustrative purpose, we consider a clinical trial powered to detect a 10% to 30% reduction in wait times on a continuous scale at the 4 different EDs with 5% of type I error rate and 80% power.

### Data Collection and Monitoring

Aggregate- and individual record-level data will be analyzed over the course of the study period to assess the effect of the intervention on patient outcomes, health service efficiency, and the cost of providing emergency care. Our analysis will include aggregate KPI data, record-level health administrative data, and aggregate financial data and will be provided to the research team by the Newfoundland and Labrador Centre for Health Information. The ED KPIs (ie, LOS, PIA, and LWBS) used as the primary outcomes for this study are further described in Table S1 in Multimedia Appendix 1 alongside other variables that will be used for statistical modeling. Each of the primary outcomes will be calculated and assessed monthly over the course of the study period. The total number of monthly ED visits will be used as the denominator for our cluster-level summaries.

Individual record-level health administrative data will include demographic variables (eg, sex, date of birth, and postal code), mortality data, wait times data, diagnosis data, and triage acuity scores, among other variables. These data will be used to create aggregate KPI and financial data provided by Newfoundland and Labrador Centre for Health Information and to assess the intervention’s impact on the type and volume of patients who visit one of the 4 selected ED sites during the study period. Financial data will capture expenditures for staff, supplies, and procedures originating from the ED and will include data related to pharmacy, diagnostic imaging, laboratory testing, surgical day care, operating room procedures, physician salaries, physician fees for service claims, nurse salaries, administrative staff salaries, ambulance services, and other related ED costs.

Patient-reported experiences and patient satisfaction survey data will be collected 3 to 5 days after ED discharge via a telephone interview conducted by a research assistant who is also an EH employee. No identifiable information related to their ED visit will be collected during the survey, and patient consent will be obtained before the interview through an implied consent process that does not require a signed consent form. We provide all study participants with the opportunity to request an informed consent document that can be provided by email or post mail and contains contact information for the principal investigator and project manager. To minimize the loss to follow-up, the research assistant will attempt to reach patients up to 3 times within 2 weeks of the initial attempt. The interview will take <30 minutes, using a questionnaire we created based on CIHI’s patient-reported experiences [38] and patient satisfaction surveys for EDs [39]. Although most of Newfoundland and Labrador’s population is English speaking (97%) [40], language was not considered an exclusion criterion. Patient interview responses will be stored via Qualtrics (a web-based survey program), where research team members will be able to analyze the data while maintaining patient anonymity. An important consideration for this approach is that the only ethically approved means of contacting patients is by telephone using patient contact information at EH. Other options were explored such as surveying patients directly in the ED, but it was deemed unsuitable, as many patients are not likely to be in a physical or mental state conducive to participating in a study.

Beyond the data provided by traditional provincial data custodians, we will also consider the SurgeCon platform’s routinely collected aggregate data. These data include calculated SurgeCon levels, ED beds, bed availability, patient acuity, and patient process tracking. The SurgeCon action-based protocol is subdivided into 5 levels of escalation and is used to indicate the level of demand, availability of resources, and capacity in the ED. The levels range from 1 (optimal operating conditions) to 5 (very busy or patient surge). They are calculated using several variables such as ED and inpatient unit bed availability, resource shortages, and number of patients in the waiting room left to be triaged or seen by a physician, among many other options. A charge nurse or other frontline staff manually enter variables used by SurgeCon’s algorithm to determine SurgeCon levels via a data entry portal. Any data entered into the data entry portal will be made accessible to the research team through the creation of a special user role that provides access to all site-specific information and the ability to export collected data.

Our implementation strategy includes the operationalization of routine data capturing by charge nurses. The research team will regularly monitor data quality and completeness across all study sites. We will be working with frontline health care staff at each of the sites to find solutions when sites are found to be missing data entry intervals or if data quality is low. We are exploring opportunities to automate data collection for certain variables if they are found to be feasible and appropriate.

### Statistical Analysis

#### Analyses

The characteristics of hospitals and patients will be recorded. We will describe the clinical and demographic characteristics of hospitals and patients for each period. We will report the response for patient satisfaction surveys and report the duration of intervention adherence at each study site and, if applicable, the reasons for noncompliance. For each outcome, we will report the results for each period, including the effect size and its precision. The 30 months of cumulatively collected data from EH and provincial health administrative databases in both the intervention and usual care periods will be modeled as a linear mixed model. We plan to analyze research data using a generalized linear mixed model (GLMM) [41] or generalized estimating equation [42]. Hussey and Hughes [43] have suggested a model-based approach for analyzing data using a repeated cross-sectional design where outcome measurements will be measured from different individuals at each measurement interval. This approach was proposed for continuous outcome variables and has been commonly used at the design stage of these studies [44]. Individuals within the same cluster are likely to be positively correlated, and the strength of the correlation can be measured by the ICC under this model and is assumed to be constant over time. However, the model suggested by Hussey and Hughes [43] has been extended to allow a more general correlation structure between individuals within the same cluster [45-47]. We will adhere to the intention-to-treat analysis; however, sensitivity analyses for comparing the results under the intention-to-treat assumption with the complier and per protocol will be conducted. Moreover, interim analyses will be conducted on a monthly basis to inform us about the findings in a timely manner and allow us to make any modifications if required [48].

The primary method of analysis used in this study is GLMM, which will consider monthly cluster-level summary measures such as means of LOS, PIA, or proportion of LWBS. Our GLMM models will include fixed effects for time, intervention effects, random effects, and random time effects for each cluster. In addition, ED setting (rural or urban), ED volume, and size of ED administrative resource will be added as cluster-level covariates. For secondary outcomes such as patient-reported experience and satisfaction, our individual-level GLMM models will include fixed effects for time, intervention effect, and random effects and random time effects for each cluster, with the covariates consisting of age, gender, the reason for ED visit, and cluster-level LOS, PIA, and proportion of LWBS.

#### Analytical Considerations

In the basic model suggested by Hussey and Hughes [43], a homogeneous secular trend is assumed across all clusters. However, this SW-CRT has numerous methodological difficulties such as confounding with time, time-varying correlation structure, change in treatment effect over time, within-cluster contamination, and change in design variation. These complexities differ according to the way SW-CRTs are designed. A summary of key methodological issues that need extra consideration when reporting SW-CRT is presented in Table S2 in Multimedia Appendix 1. We will be continuously monitoring and intervening when necessary to manage analytical challenges associated with this kind of design. Any unexpected event during the study period will be recorded, and methodological approaches to overcome the issue will be described in the reports.

### Patient Engagement

As a patient-oriented study, the inclusion of patients in the research team is critical. An example of this is our lead patient research partner who has been advising the research team since the pilot study. The research team is also advised by the patient engagement working group, which comprises the lead patient partner, scientific patient engagement lead, clinicians, researchers, and students. We designed the protocol to give patients a variety of opportunities to participate in research activities. Applicable research activities vary in terms of the level of engagement required (eg, surveys vs full-team membership). We are currently recruiting additional patient research partners for the study’s patient engagement working group, but patients can also be involved in other committees and working groups suited to their interests and needs. Overall, the level of engagement will vary from receiving information to consultation to full collaboration with patient partners to inform all project activities. We used 4 essential pillars, as suggested by Shippee et al [49], to inform our patient engagement strategy, which is further described in our implementation protocol [24]. We are committed to upholding the guiding principles of inclusiveness, support, mutual respect, and cobuilding inherent in these pillars.

### Impact of COVID-19

The impact of the COVID-19 pandemic on the study and its protocol is ongoing. Public health care measures such as stay-at-home orders, handwashing, and physical and social distancing compounded with COVID-19 concerns have caused rapid disruptions in daily life and a delay in the implementation of the intervention. A notable effect may be a significant change in the number of patients visiting EDs. The fear that the public is experiencing because of COVID-19 is likely exacerbated by measures related to stay-at-home orders, self-isolation protocols, including quarantines, travel restrictions, and closures of nonessential businesses. With most of Newfoundland and Labrador’s residents practicing social distancing and self-isolating and an increase in the number of workers who are now working remotely, the potential for injuries such as trauma due to motor vehicle collisions may decline considerably [50]. Given the potential reduction in injury and the climate of fear at the beginning of the COVID-19 pandemic, patients were found to be less likely to use hospital ED services [51]. Another impact of the pandemic is the temporary suspension of essential and nonessential medical procedures. The suspension of certain health care services may have downstream effects on wait times because delays in surgeries and other important medical procedures could increase the number of higher acuity patients who develop complications because of the delay. These higher acuity patients require additional resources and time and can have a significant impact on patient flow and wait times. Other pandemic-related considerations include added wait times because of public health measures such as increased handwashing, social distancing, donning and doffing of personal protective equipment, and sanitizing high-touch surfaces. More recently, a transition from in-person to web-based or telephone family physician consultations may be increasing ED patient volumes [52,53]. The pandemic has also exacerbated issues related to physician and nursing shortages in the province [54-56]. As a result, achieving optimal sample size from each of the ED sites might be compromised because of pandemic-related ED service use trends. The research team will look to explore the effects of the COVID-19 pandemic on patient flow and wait times in EDs.

### Description of Intervention

**Figure 3:**
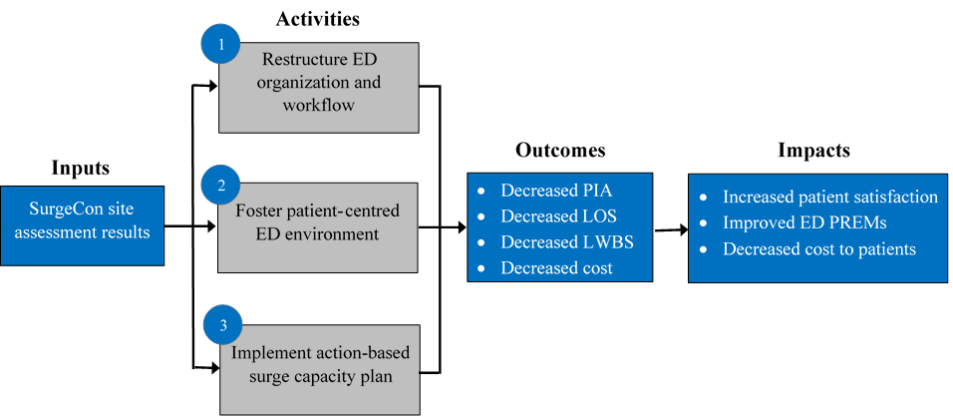
The SurgeCon intervention logic model. ED: emergency department; LOS: length of stay; LWBS: leaving the emergency department without being seen; PIA: physician’s initial assessment; PREM: patient-reported experience measure.

Our intervention comprises several components that will be implemented sequentially. The SurgeCon intervention is a pragmatic ED management platform that includes 3 distinct intervention components that together act to improve ED efficiency, patient satisfaction, and the value of emergency service costs (Table 1). SurgeCon’s intervention process starts with a site assessment during the transition period that aims to clarify key performance issues, collect information related to the ED’s organizational and workflow structures, and prepare ED staff and management for upcoming operational changes, while also establishing a patient-centered ED environment and action-based ED management (Figure 3). Due to the high degree of variability that exists between EDs, the information collected during the site assessment allows for the customization of SurgeCon’s underlying protocols and determines whether certain components of the intervention are appropriate or applicable for implementation. A site assessment will be conducted by members of our working group who developed SurgeCon in Carbonear. The implementation working group will follow a 4-step protocol for these site assessment visits; for further details please see the implementation protocol [24].

**Table 1:** Intervention components and their associated action plans.

Intervention components and action plans		Description and strategy
**Restructure ED^a^ organization and workflow**		
	Stable patient priority setting	Canadian EDs have been using the CTAS^b^ since 1999 [57]. What the SurgeCon intervention proposes is to augment CTAS by combining CTAS scores and waiting times to best meet the suggested time frame set out by the CTAS organization. Patients who are CTAS level 1 (resuscitation) or 2 (emergent) who are not stable will still be treated immediately in this model, but individuals who are less urgent (CTAS 3-5) will be treated when the CTAS 1-2 individuals become more stable, but before they are discharged. This allows the physician to quickly treat and discharge less urgent individuals before returning to spend more time with the urgent cases. This prioritization method can significantly improve patient flow without having to compromise patient safety.
	Door-to-door focus	A number of studies have found a strong correlation between patient satisfaction and PIA^c^; the shorter the PIA, the more satisfied the patient [58,59]. To reduce the time to PIA, we will use the following strategies: ED physicians and frontline providers will triage with nursing staff with the goal of increased patient discharge from the fast-track area or triage room without waiting. Triage nurse–driven orders (eg, symptom management, laboratory testing, and diagnostic imaging) will only be applied on patients who would be waiting longer than 1 hour to see a physician. If the patient can be seen by a physician within an hour, waiting for potentially unnecessary test results could delay the PIA. ED physicians will review patients arriving on ambulance stretchers in the hallway if there are no available beds instead of waiting for a bed to be free.
	Nurse practitioner–physician communication	SurgeCon reorganizes the traditional Canadian ED communication structure by promoting communication between NPs^d^, ERPs^e^, and RNs^f^ to work collaboratively to improve patient flow through appropriate allocation of patients. By opening lines of communication between NPs, ERPs, and RNs, the entire ED can work in unison to move patients further along their path to being discharged.
**Establish a patient-centered ED environment**		
	Improve the overall appearance of physical spaces in the ED (eg, waiting room, fast-track zone, examination rooms, and treatment space) to improve patient satisfaction	This will be conducted in collaboration with Eastern Health. In consultation with our local patient partners, we will renovate, redecorate, and declutter ED spaces, removing outdated or irrelevant wall postings. All subsequent wall postings will require departmental approval and will be placed in a central location.
**eHealth action–based ED management**		
	eHealth ED management solution	Using automated extraction of data from HISs^g^ where possible and manual entry otherwise, SurgeCon’s digital component will be able to perform real time analysis on extracted data in a routine and timely manner to give ED staff a sense of overall demand and available resources at any given time. SurgeCon’s eHealth component will be installed and tested in each hospital during the adoption phase of the study. In situations where data elements might not be captured by existing ED repositories, an ED staff member will address this issue by manually entering specific variables (eg, number of ambulances waiting to be off-loaded) as part of their regular duties. Manual entry occurs via a web-based SurgeCon portal and is subsequently reported at a frequency that is determined by ED staff and management. SurgeCon’s eHealth component is currently available on desktops and mobile devices and has been deployed in Eastern Health’s secure network with the assistance of the Newfoundland and Labrador Centre for Health Information. The data entry portal and dashboard that provides real time data is normally displayed on a large digital whiteboard in close proximity to the nursing station. This allows all team members to have a clear understanding of the level of demand, current capacity, and available resources. The Carbonear pilot site has since operationalized the task of a 2-hour data entry interval and have done so without a significant workforce change. We have found that a 2-hour interval for data entry is a feasible target and can be quickly performed once certain reporting processes have been established [30,31]. We have further developed and tested the digital application at our pilot site. The development of the application will continue throughout the study to ensure feedback and information are incorporated into iterative software updates.
	eHealth action–based protocol	We have created a unique frontline, action-based protocol that helps ED staff (paramedics, nurses, and physicians) manage their actions to actively reduce patient surges and wait times and increase patients’ access to emergency medical care. The protocol is delivered via a digital whiteboard app, which will be installed in the nursing station in the ED. The app uses algorithms (adjusted to meet the needs of each hospital) to advise when to use volume-based staffing (shifting staff between areas of the hospital based on workload), appropriate and timely involvement of hospital management, and overcapacity protocols, which may otherwise be overlooked by distracted frontline ED staff. All intervention sites will be routinely collecting data related to staff availability, ED and inpatient bed availability, aggregate patient acuity, and process tracking, among other important variables through SurgeCon’s eHealth component. A SurgeCon level is calculated via an algorithm that uses variable data to determine the level of demand in the department and resource availability to meet the demand. The action-based protocol included in SurgeCon’s eHealth platform assigns actions based on the SurgeCon level calculated. The following list includes examples of actions that may be assigned once a threshold for a specific variable has been exceeded: Observation: Patients admitted in ED Action: Notify charge nurse on accepting unit to create a plan for timely transfer of admissions Observation: Critical patients (1:1 nursing care) Action: Notify ICU^h^ to plan for help Observation: Pending transfer out Action: If the flight team requires it, make appropriate arrangements

^a^ED: emergency department.

^b^CTAS: Canadian Triage and Acuity Scale.

^c^PIA: physician’s initial assessment.

^d^NP: nurse practitioner.

^e^ERP: emergency room physician.

^f^RN: registered nurse.

^g^HIS: hospital information system.

^h^ICU: intensive care unit.

### Intervention Components

The SurgeCon intervention is guided by continuous process improvement principles that look to ultimately improve quality of services, reduce waste (low-value care), reduce time (ED wait times), and reduce cost (eg, cost per patient, cost of overtime, and cost to patients) [60-63]. The following components of the SurgeCon intervention will follow these principles closely and will be implemented using other continuous improvement methods such as *Kaizen* events [64]. The SurgeCon intervention includes 3 components (Figure 3).

The major intervention components and their action plans with description and strategy are given in Table 1.

## Results

This study was funded in April 2019, was approved by the Newfoundland and Labrador Health Research Ethics Board on March 19, 2020, and is now registered with ClinicalTrials.gov. Data collection for this study is ongoing. As of February 2022, a total of 570 randomly selected patients have participated in telephone interviews focusing on patient-reported experiences and patient satisfaction with ED wait times. The first intervention site was randomly selected and began intervention implementation in September 2021. Since that time, SurgeCon’s eHealth component has been configured, and deployed and health care staff have received ED patient flow training. The second intervention site is scheduled to be randomly selected and begin intervention implementation during the winter of 2022.

## Discussion

### Principal Findings

The SurgeCon intervention has the potential to be a scalable solution that can address ED wait times. In this paper, we have described the protocol for a stepped wedge design, which is a relatively new type of study design that is progressively being used to evaluate the efficiency of public health services. This study design is known for its application in assessing the implementation of evidence-based quality improvement initiatives in health care settings. We chose the stepped wedge design as an informative, efficient, and valid design to examine the SurgeCon platform’s effectiveness in improving important patient and health service processes and outcomes in EDs located in a single region of Newfoundland and Labrador.

### Conclusions

By assessing the impact of the SurgeCon intervention on the efficiency of ED services, we hope to be able to improve wait times and patient experiences and produce better-value care.

## Appendix

Multimedia Appendix 1 Definitions, sources, and purpose of study variables; methodological issues encountered; and applicable strategies to overcome them.

## Abbreviations

CIHI: Canadian Institute for Health Information
ED: emergency department
EH: Eastern Health
GLMM: generalized linear mixed model
ICC: intracluster correlation coefficient
KPI: key performance indicator
LOS: length of stay
LWBS: leaving the emergency department without being seen
PIA: physician’s initial assessment
RE-AIM: reach, effectiveness, adoption, implementation, and maintenance
RHA: regional health authority
SW-CRT: stepped wedge cluster randomized trial

## References

[1] Torjesen I (2018). Latest waiting time figures for emergency departments in England are worse on record. BMJ.

[2] (2018). Emergency department wait times in Canada continuing to rise. Canadian Institute for Health Information.

[3] (2018). Dramatic rise in waiting times at emergency departments. BBC News.

[4] Katz A, Enns J (2018). How long is too long to wait in an emergency room?. Huffington Post.

[5] (2010). The commonwealth fund 2010 international health policy survey in eleven countries. The Commonwealth Fund.

[6] Vezyridis P, Timmons S (2014). National targets, process transformation and local consequences in an NHS emergency department (ED): a qualitative study. BMC Emerg Med.

[7] Chang AM, Lin A, Fu R, McConnell KJ, Sun B (2017). Associations of emergency department length of stay with publicly reported quality-of-care measures. Acad Emerg Med.

[8] Barker J (2018). Labrador senior waits 10 hours in emergency room without care for broken arm. CBC News.

[9] Hutton F (2018). Picture of senior waiting for hours in St.John's emergency room sparks outrage. CBC News.

[10] Breen K (2018). St. Anthony patients kept on stretchers while beds remained in storage. CBC News.

[11] Carter AJ, Chochinov AH (2007). A systematic review of the impact of nurse practitioners on cost, quality of care, satisfaction and wait times in the emergency department. CJEM.

[12] Maggio PM, Brundage SI, Hernandez-Boussard T, Spain DA (2009). Commitment to COT verification improves patient outcomes and financial performance. J Trauma.

[13] Jarvis PR (2016). Improving emergency department patient flow. Clin Exp Emerg Med.

[14] Schroeppel TJ, Sharpe JP, Magnotti LJ, Weinberg JA, Croce MA, Fabian TC (2014). How to increase the burden on trauma centers: implement the 80-hour work week. Am Surg.

[15] Schroeppel TJ, Sharpe JP, Magnotti LJ, Weinberg JA, Croce MA, Fabian TC (2015). How to further decrease the efficiency of care at a level I trauma center: implement the amended resident work hours. Am Surg.

[16] Christmas E, Johnson I, Locker T (2013). The impact of 24 h consultant shop floor presence on emergency department performance: a natural experiment. Emerg Med J.

[17] Han JH, Zhou C, France DJ, Zhong S, Jones I, Storrow AB, Aronsky D (2007). The effect of emergency department expansion on emergency department overcrowding. Acad Emerg Med.

[18] Mumma BE, McCue JY, Li CS, Holmes JF (2014). Effects of emergency department expansion on emergency department patient flow. Acad Emerg Med.

[19] van Veelen MJ, van den Brand CL, Reijnen R, van der Linden MC (2016). Effects of a general practitioner cooperative co-located with an emergency department on patient throughput. World J Emerg Med.

[20] Morin C, Choukroun J, Callahan JC (2018). Safety and efficiency of a redirection procedure toward an out of hours general practice before admission to an emergency department, an observational study. BMC Emerg Med.

[21] (2020). Hospital spending: focus on the emergency department. Canadian Institute for Health Information.

[22] (2016). Eastern health's annual performance report 2015-2016. Eastern Health.

[23] (2012). A strategy to reduce emergency department wait times in Newfoundland and Labrador. Health and Community Services, Government of Newfoundland.

[24] Anaraki NR, Jewer J, Hurley O, Mariathas H, Young C, Norman P, Patey C, Wilson B, Etchegary H, Senior D, Ashgari S (2021). Implementation of an ED surge management platform: a study protocol. Research Square (forthcoming).

[25] Glasgow RE, Harden SM, Gaglio B, Rabin B, Smith ML, Porter GC, Ory MG, Estabrooks PA (2019). RE-AIM planning and evaluation framework: adapting to new science and practice with a 20-year review. Front Public Health.

[26] Damschroder LJ, Aron DC, Keith RE, Kirsh SR, Alexander JA, Lowery JC (2009). Fostering implementation of health services research findings into practice: a consolidated framework for advancing implementation science. Implement Sci.

[27] Barker D, McElduff P, D'Este C, Campbell MJ (2016). Stepped wedge cluster randomised trials: a review of the statistical methodology used and available. BMC Med Res Methodol.

[28] Beard E, Lewis JJ, Copas A, Davey C, Osrin D, Baio G, Thompson JA, Fielding KL, Omar RZ, Ononge S, Hargreaves J, Prost A (2015). Stepped wedge randomised controlled trials: systematic review of studies published between 2010 and 2014. Trials.

[29] Brown CA, Lilford RJ (2006). The stepped wedge trial design: a systematic review. BMC Med Res Methodol.

[30] Asghari S (2021). SurgeCon: an emergency department surge management platform. Clinical Trials.

[31] Patey C, Asghari S, Norman P, Hurley O (2020). Redesign of a rural emergency department to prepare for the COVID-19 pandemic. CMAJ.

[32] Patey C, Norman P, Araee M, Asghari S, Heeley T, Boyd S, Hurley O, Aubrey-Bassler K (2019). SurgeCon: priming a community emergency department for patient flow management. West J Emerg Med.

[33] Killip S, Mahfoud Z, Pearce K (2004). What is an intracluster correlation coefficient? Crucial concepts for primary care researchers. Ann Fam Med.

[34] Heaton HA, Castaneda-Guarderas A, Trotter ER, Erwin PJ, Bellolio MF (2016). Effect of scribes on patient throughput, revenue, and patient and provider satisfaction: a systematic review and meta-analysis. Am J Emerg Med.

[35] Bellamkonda VR, Kumar R, Scanlan-Hanson LN, Hess JJ, Hellmich TR, Bellamkonda E, Campbell RL, Hess EP, Nestler DM (2016). Pilot study of Kano "Attractive Quality" techniques to identify change in emergency department patient experience. Ann Emerg Med.

[36] Griffen D, Callahan CD, Markwell S, de la Cruz J, Milbrandt JC, Harvey T (2012). Application of statistical process control to physician-specific emergency department patient satisfaction scores: a novel use of the funnel plot. Acad Emerg Med.

[37] Boudreaux ED, Mandry CV, Wood K (2003). Patient satisfaction data as a quality indicator: a tale of two emergency departments. Acad Emerg Med.

[38] (2015). PROMS: background document. Canadian Institute for Health Information.

[39] (2018). Patient experience. Canadian Institute for Health Information.

[40] (2016). Language highlight tables, 2016 Census. Statistics Canada.

[41] McCullagh P, Nelder JA (1989). Generalized linear models. 2nd edition.

[42] Jiang J (1998). Consistent estimators in generalized linear mixed models. J Am Stat Assoc.

[43] Hussey MA, Hughes JP (2007). Design and analysis of stepped wedge cluster randomized trials. Contemp Clin Trials.

[44] Martin J, Taljaard M, Girling A, Hemming K (2016). Systematic review finds major deficiencies in sample size methodology and reporting for stepped-wedge cluster randomised trials. BMJ Open.

[45] Girling AJ, Hemming K (2016). Statistical efficiency and optimal design for stepped cluster studies under linear mixed effects models. Stat Med.

[46] Hooper R, Teerenstra S, de Hoop E, Eldridge S (2016). Sample size calculation for stepped wedge and other longitudinal cluster randomised trials. Stat Med.

[47] Kasza J, Hemming K, Hooper R, Matthews J, Forbes AB (2019). Impact of non-uniform correlation structure on sample size and power in multiple-period cluster randomised trials. Stat Methods Med Res.

[48] Kumar A, Chakraborty BS (2016). Interim analysis: a rational approach of decision making in clinical trial. J Adv Pharm Technol Res.

[49] Shippee ND, Domecq Garces JP, Prutsky Lopez GJ, Wang Z, Elraiyah TA, Nabhan M, Brito JP, Boehmer K, Hasan R, Firwana B, Erwin PJ, Montori VM, Murad MH (2015). Patient and service user engagement in research: a systematic review and synthesized framework. Health Expect.

[50] Kamine TH, Rembisz A, Barron RJ, Baldwin C, Kromer M (2020). Decrease in trauma admissions with COVID-19 pandemic. West J Emerg Med.

[51] (2021). COVID-19’s impact on emergency departments. Canadian Institute of Health Information.

[52] (2021). Covid-19 update regarding the provision of in-person and virtual care. The College of Physicians and Surgeons of Ontario.

[53] (2021). COVID-19 update re: in-person and virtual care. College of Physicians and Surgeons of British Columbia.

[54] (2021). Registered nurses' union calls on parties to address growing shortage of registered nurses. Registered Nurses' Union.

[55] Physicians in NL stand in solidarity with our registered nurse colleagues on their day of action. Newfoundland and Labrador Medical Association (NLMA) News.

[56] (2019). Report: physician resource forecast for family medicine: Newfoundland and Labrador. Health Intelligence Inc.

[57] (2012). Health care in Canada, 2012: a focus on wait times. Canadian Institute for Health Information.

[58] El Sayed MJ, El-Eid GR, Saliba M, Jabbour R, Hitti EA (2015). Improving emergency department door to doctor time and process reliability: a successful implementation of lean methodology. Medicine (Baltimore).

[59] Pang PS, McCarthy D, Schmidt M, Weiner M, Gavran G, Patterson B, Lee T, Lucenti MJ (2011). 440 Factors associated with perfect Press Ganey satisfaction scores for discharged emergency department patients. Ann Emerg Med.

[60] Womack JP, Jones DT, Roos D (1990). The machine that changed the world: the story of lean production--Toyota's secret weapon in the global car wars that is now revolutionizing world industry.

[61] Holweg M (2007). The genealogy of lean production. J Oper Manag.

[62] Poole T, Mazur LM (2010). Assessing readiness for lean change in emergency department. Proceedings of the 2010 Industrial Engineering Research Conference.

[63] Vashi AA, Sheikhi FH, Nashton LA, Ellman J, Rajagopal P, Asch SM (2019). Applying lean principles to reduce wait times in a VA emergency department. Mil Med.

[64] (2018). Kaizen. Lean production.

