# Supplementary material for: A Quality Improvement Emergency Department Surge Management Platform (SurgeCon): Protocol for a Stepped Wedge Cluster Randomized Trial
Source: JMIR Res Protoc. 2022 Mar 24;11(3):e30454. doi: 10.2196/30454 (PMC8990381; doi:10.2196/30454)
Supplement: Multimedia Appendix 1 [file resprot_v11i3e30454_app1.docx]

**APPENDIX**

Table S1: Definitions, sources, and purpose of study variables

| **Variables** | **Definition** | **Source** | **Purpose** |
| --- | --- | --- | --- |
| **Outcome Variables** | | | |
| ED Level | | | To describe and compare differences before and after SurgeCon implementation. |
| Length of Stay for Departed Patients | Time (in minutes) from the first recorded encounter (triage or registration) to discharge from the ED (in minutes). ED level average will be calculated on monthly basis | Medico-administrative data |  |
| Physician Initial Assessment | Time (in minutes) from the first recorded encounter (triage or registration) to first assessment by a physician or their delegate (i.e. trainee, physician assistant, or nurse practitioner). ED level average will be calculated on monthly basis | Medico-administrative data |  |
| Left Without Being Seen | Percentage of patients who leave the ED before being assessed by a physician or their delegate (i.e. trainee, physician assistant, or nurse practitioner. ED level average will be calculated on monthly basis | Medico-administrative data |  |
| Cost (see detailed cost variables below) | Cost of providing services in ED | Provincial Management Information System (MIS) Financial Data |  |
| Patient Level | | | To describe and compare differences before and after SurgeCon implementation. |
| Satisfaction | The extent to which a person is happy with the healthcare services he/she received from their most recent ED visit | Survey |  |
| Patient reported experience of ED service | Patient feedback on the quality of care they experienced during their most recent ED visit | Survey |  |
| Cost | Cost of ED visit to patient | Cost questionnaire |  |
| **Other Variables** | | | |
| Hospital Facility ID | Hospital Code | Medico-administrative data | To define hospital level data |
| Patient ID | De-identified ID | Medico-administrative data/survey | To define individual level data |
| Sex | Sex (male, female) | Medico-administrative data/survey | To describe/ compare groups/subgroup analysis |
| Age | Birth year | Medico-administrative data/survey |  |
| CTAS score | Level of acuity of patient who visited ED | Medico-administrative data |  |
| Discharge Disposition | Discharge status (eg, Transfer, death, etc.) | Medico-administrative data | To validate / define reported aggregate ED wait time metrics. |
| Arrival Date/Time | Patient arrival time | Medico-administrative data |  |
| Registration Date/Time | Patient registration time | Medico-administrative data |  |
| Time to Treatment by Treatment type | Amount of time required to receive a specific treatment (eg, antibiotics, thrombolysis, analgesic) | Medico-administrative data |  |
| ER Departure Date/Time | Patient discharge, transfer, departed time | Medico-administrative data |  |
| Decision to Admit Date and Time | Patient admission decision time | Medico-administrative data |  |
| **Adverse Effect Variables** | | | |
| Mortality | Number of patients who died in a specific time (7-day, 30-day, 1 year) period after visiting the ED. | Medico-administrative data | To determine whether SurgeCon is adversely affecting patient safety or ED performance. |
| Unscheduled/Unplanned  Returns | Proportion of patients returning to the ED unplanned within a specific period of time (24 hours/72 hours) | Medico-administrative data |  |
| Hospital Readmission Rate | Number of patients readmitted over a 24-hour period. | Medico-administrative data |  |
| Hospital Occupancy Rate | Proportion of patients relative to the number of beds available at the hospital (24-hour rate). | Medico-administrative data |  |
| **Cost Variables** | | | |
| Laboratory | Number and type of laboratory tests originating from the ED | Provincial MIS Financial Data | To estimate cost of ED operations. |
| Pharmacy | Number and type of prescriptions originating from the ED | Provincial MIS Financial Data |  |
| Radiology | Number and type of diagnostic radiology tests originating from the ED | Provincial MIS Financial Data |  |
| Catheterization | Number of catheterizations originating from the ED | Provincial MIS Financial Data |  |
| Operating room | Number and type of surgeries originating from the ED | Provincial MIS Financial Data |  |
| Other ED related costs | Other costs associated with normal ED operations (eg, Maintenance, housekeeping, supplies) | Provincial MIS Financial Data |  |
| ED Physician | Physicians’ salary expenditures for both primary and secondary physicians | Provincial MIS Financial Data |  |
| ED Nurse Admin | Nurse Admin salary expenditures | Provincial MIS Financial Data |  |
| ED Nurse Practitioner | Nurse Practitioner salary expenditures | Provincial MIS Financial Data |  |
| ED Nursing/Paramedic | Salary expenditures for nursing staff and paramedics- this includes all payment types (Regular, Premium, OT, Relief, Contribution, Other) | Provincial MIS Financial Data |  |
| ED Receptionist | Receptionist salary expenditures | Provincial MIS Financial Data |  |
| Ambulance services | Cost of providing ambulance services | Provincial MIS Financial Data |  |

Table S2: Methodological issues encountered and applicable strategies to overcome them

| Concern | Description | Importance | Alternative Strategy |
| --- | --- | --- | --- |
| Imbalance design | In SW-CRT, different clusters are sequentially randomized to different time points. With respect to time, measurements that are observed under the control arm are from an earlier calendar time than the intervention arm. | Intervention and outcome measurements are associated with the time which is likely to be a confounder. | The intra-cluster correlation and time effect that were estimated from the fitted model will be recorded for use in the design of future trials and will allow any underlying confounding effects of calendar time for analysis. |
| Repeated measurements collected from the same participants at the same site | A series of measurements will be observed over time within each cluster. These measurements can either be for the same or different participant or a mixture of both. | The correlation structure of the design is complicated when compared to a traditional CRT. | Both analysis and sample size calculations will allow for the fact that data are not independent and dependencies might vary over time. We account for within-period ICCs as well as between-period ICCs. |
| Site contamination | Some or all of the ED sites will be exposed to both the intervention and control conditions. ED staff can either have a relatively short exposure to the SurgeCon intervention or a long exposure. | It is unlikely that individuals will be exposed to both the control and intervention conditions when the duration of exposure is short but possible for some individuals to be exposed to both arms when the duration of exposure is long. | We will hold separate training sessions for each ED. We will also ask the ED teams not to use/share knowledge obtained through intervention related activities if they are serving in the control arm of another site. In addition, we will compare the changes within and between sites when they do receive the intervention. |
| Delayed treatment effects | The possible immediate effect or possible delay effect of the SurgeCon intervention should be taken into account. | To have a better estimate of intervention effectiveness, we have introduced a transition period to avoid a delay effect. | Our trial has a one month period for training frontline staff and implementing other intervention components, which occurs during the transition period. Training and intervention adherence will continue until the end of the study period. Moreover, we will conduct interim analysis to identify any delay effect. |
| Interaction effect between time and treatment | Research activities related to the implementation of the intervention may need to be repeated if long lasting impacts and intervention adoption are not observed. The intervention itself is also subject to change since this trial design allows for modifications to be made based on feedback and data analysis. | The impact of implementing SurgeCon at an intervention site might progressively change with time. We may expect a change in the effect produced by the intervention if the intervention itself changes over time. | Our analysis will take into consideration any modifications made to the intervention and whether the modifications or the originally designed intervention are responsible for observed changes in department efficiency. |
| Sampling of observations | Participants are recruited on a continuous basis as they visit one of the four intervention sites during the study period. They can also be randomly selected for patient satisfaction and experience telephone interviews. | There is a risk of bias related to the sampling strategy used to collect data. | Each site will be monitored by a site coordinator for data collection. A statistician who is a member of the iCT working group will supervise sampling at the four study sites. The iCT working group will provide advice on methodological best practices. |
| Continuous or discrete time measurements | Measurements can be collected in the form of continuous (eg, patients ED wait time), or discrete (eg, patient satisfaction survey questionnaire collected at some discrete points in time). | Observations that are collected continuously are likely to be measured in a continuous fashion whereas, outcomes that are collected in discrete time, are more likely to be observed in a discrete manner. | Continuous outcome of KPI and survey data will be analyzed by considering time as continuous and discrete time points respectively. A site coordinator will monitor data collection in each site and record any unexpected event. The analysis will be revisited if any unexpected event is reported. |
